# The Effect of Obstructive Sleep Apnea Surgery on Oxidative Stress Markers: A Prospective Observational Study

**DOI:** 10.3390/jcm15114204

**Published:** 2026-05-29

**Authors:** Ülkü İnce, Hanife K. Kabukcu, Aslı Bostancı, Sebahat Ozdem, Murat Turhan, Ikbal O. Kucukcetın

**Affiliations:** 1 Department of Anesthesiology and Reanimation, Erzurum City Hospital, Erzurum 25070, Türkiye; 2 Department of Anesthesiology and Reanimation, Akdeniz University Faculty of Medicine Hospital, Antalya 07070, Türkiye; hanifekabukcu@akdeniz.edu.tr; 3 Department of Otorhinolaryngology, Akdeniz University Faculty of Medicine Hospital, Antalya 07070, Türkiye; draslibostanci@gmail.com (A.B.); muratturhan@akdeniz.edu.tr (M.T.); 4 Department of Biochemistry, Akdeniz University Faculty of Medicine Hospital, Antalya 07070, Türkiye; ozdem@akdeniz.edu.tr (S.O.); ikbalozen@akdeniz.edu.tr (I.O.K.)

**Keywords:** obstructive sleep apnea syndrome, oxidative stress, antioxidant, protein oxidation, surgery

## Abstract

**Background:** This study aimed to evaluate the effects of obstructive sleep apnea syndrome (OSAS) surgery on oxidative stress, antioxidant capacity, and oxidative protein modifications. Changes in Total Oxidant Status (TOS), Total Antioxidant Status (TAS), Advanced Oxidation Protein Products (AOPP), Ischemia-Modified Albumin (IMA), and Total Thiol (TT) levels were analyzed in the preoperative and postoperative periods. **Methods:** Sixty-seven patients aged 18–65 years with an Apnea–Hypopnea Index (AHI) > 5 who underwent OSAS surgery were included. Demographic characteristics were recorded. Venous blood samples were collected at four time points: before anesthesia induction (T1), at the end of surgery (T2), on postoperative day 3 (T3), and at postoperative month 3 (T4). TOS, TAS, AOPP, IMA, and TT levels were measured and statistically evaluated. **Results:** Of the patients, 13.4% (n = 9) were female and 86.6% (n = 58) were male. No significant changes were observed in TOS, TAS, AOPP, or IMA levels between time points. However, TT levels at postoperative month 3 were significantly higher than those at earlier measurements (*p* < 0.05). Positive correlations were found between AHI and TOS, AOPP, and IMA levels, while TAS and TT showed no significant correlation with AHI. **Conclusions:** Surgery for OSAS did not produce significant postoperative improvements in oxidative stress parameters except for TT elevation. These results indicate that surgical intervention alone may be insufficient for biochemical recovery in OSAS and should be complemented by lifestyle and supportive therapies.

## 1. Introduction

Obstructive Sleep Apnea Syndrome (OSAS) is a highly prevalent and increasingly recognized public health problem characterized by recurrent episodes of partial or complete obstruction of the upper airway during sleep [1,2]. These episodes lead to intermittent hypoxia, hypercapnia, sleep fragmentation, and significant fluctuations in intrathoracic pressure. Although traditionally considered a disorder limited to sleep disruption and daytime somnolence, OSAS is now widely acknowledged as a systemic disease with substantial multisystem involvement.

The pathophysiological consequences of OSAS extend far beyond the respiratory system. Recurrent hypoxia–reoxygenation cycles trigger a cascade of biological responses, including sympathetic nervous system activation, endothelial dysfunction, metabolic dysregulation, and systemic inflammation. These processes contribute to the development of major comorbid conditions such as hypertension, coronary artery disease, heart failure, insulin resistance, type 2 diabetes mellitus, and cerebrovascular disorders [3,4]. In addition, OSAS has been associated with impaired cognitive performance, mood disorders, and decreased quality of life, further emphasizing its clinical significance.

Among the mechanisms underlying these systemic effects, oxidative stress plays a central and unifying role. Oxidative stress arises when the production of reactive oxygen species (ROS) exceeds the capacity of antioxidant defense systems to neutralize them. In OSAS, intermittent hypoxia acts as a potent stimulus for ROS generation, particularly through mitochondrial dysfunction and the activation of enzymatic sources such as NADPH oxidase. The repetitive cycles of hypoxia and reoxygenation resemble ischemia–reperfusion injury, resulting in oxidative damage to cellular components, including lipids, proteins, and nucleic acids. Over time, this oxidative burden contributes to vascular injury, inflammation, and progressive organ dysfunction.

The evaluation of oxidative stress in clinical studies relies on a range of biomarkers that collectively reflect the balance between oxidant production and antioxidant capacity. Total Oxidant Status (TOS) provides a measure of the overall oxidant load in the circulation, while Total Antioxidant Status (TAS) reflects the cumulative activity of antioxidant systems. In addition to these global markers, more specific indicators of oxidative protein damage are increasingly used. Advanced Oxidation Protein Products (AOPP) represent dityrosine-containing, cross-linked protein products formed during oxidative stress, whereas Ischemia-Modified Albumin (IMA) reflects structural alterations in albumin caused by ischemia and oxidative injury. Total Thiol (TT), which measures sulfhydryl-containing compounds, is an important component of the antioxidant defense system and plays a key role in maintaining redox homeostasis.

The management of OSAS aims to eliminate airway obstruction, improve oxygenation, and reduce long-term complications. Continuous Positive Airway Pressure (CPAP) therapy remains the gold standard treatment; however, its effectiveness is often limited by poor patient adherence. Alternative therapeutic strategies include oral appliances, positional therapy, lifestyle modifications such as weight reduction and increased physical activity, and pharmacological interventions. In selected patients, particularly those with anatomical abnormalities or intolerance to CPAP, surgical treatment is considered a viable option. Surgical approaches such as uvulopalatopharyngoplasty, septoplasty, and tongue base procedures aim to increase airway patency and reduce collapsibility during sleep [5,6].

Despite the established clinical benefits of surgical interventions in improving airway function and symptomatology, their impact on systemic biochemical processes—particularly oxidative stress—remains insufficiently understood. Most existing research has focused on the effects of CPAP therapy on inflammatory and oxidative markers, with inconsistent and sometimes conflicting results [7,8,9,10]. In contrast, studies evaluating the biochemical outcomes of OSAS surgery are limited in number and scope. İnce et al. examined arylesterase, paraoxonase, and nitrotyrosine levels as antioxidant biomarkers after OSAS surgery [11]. No significant changes were observed in these parameters after OSAS surgery. In this study, we aimed to provide a more comprehensive biochemical assessment of oxidative stress and antioxidant balance in the perioperative period.

Given the central role of oxidative stress in OSAS pathogenesis and its contribution to disease-related complications, evaluating changes in oxidative markers following surgical treatment is of considerable clinical importance. Understanding whether surgical correction of airway obstruction can reverse or mitigate oxidative imbalance may provide insight into its potential to reduce long-term systemic risk.

Despite the growing evidence linking OSAS to systemic oxidative stress, the biochemical consequences of surgical treatment remain insufficiently characterized. Most previous studies have primarily focused on clinical outcomes such as symptom relief, apnea–hypopnea index reduction, and quality-of-life improvement, while perioperative oxidative and antioxidant responses have received comparatively less attention. Furthermore, oxidative stress markers may provide additional insight into the systemic effects of surgery beyond conventional polysomnographic parameters. Evaluating these biochemical alterations may contribute to a better understanding of whether surgical treatment can reverse the oxidative burden associated with intermittent hypoxia and chronic inflammation in OSAS. Therefore, a detailed investigation of oxidative stress pathways may help clarify the biological mechanisms underlying postoperative recovery and identify potential biomarkers for treatment monitoring.

Therefore, the present study aimed to comprehensively evaluate perioperative changes in TOS, TAS, AOPP, IMA, and TT levels in patients undergoing surgical treatment for OSAS. By assessing both global oxidative status and specific markers of protein oxidation, this study seeks to clarify the biochemical impact of surgical intervention and contribute to a more comprehensive understanding of OSAS management.

## 2. Materials and Methods

### 2.1. Study Design and Ethical Considerations

This prospective observational study was conducted at the Department of Otorhinolaryngology, Akdeniz University Faculty of Medicine. Ethical approval was obtained from the institutional ethics committee, and all procedures were performed in accordance with the Declaration of Helsinki. Written informed consent was obtained from all participants.

### 2.2. Sample Size and Participants

Due to the absence of prior studies evaluating these specific biomarkers perioperatively, sample size calculation was performed using G*Power 3.1 software. Based on repeated-measures ANOVA with an effect size of 0.25, α = 0.05, and a power of 0.95, a minimum of 36 patients was required. To enhance reliability, 67 patients were ultimately included.

Inclusion criteria were age 18–65 years, American Society of Anesthesiologists (ASA) physical status I–II, and Apnea-Hypopnea Index (AHI) > 5. Exclusion criteria included ASA III–IV, chronic inflammatory diseases, smoking, and postoperative complications.

### 2.3. Surgical Procedures and Follow-Up

Patients underwent various surgical procedures depending on anatomical findings, including septoplasty, uvulopalatopharyngoplasty, tongue base resection, and tongue base suspension. All surgeries were performed under standardized anesthesia protocols.

Postoperatively, patients were monitored in the Postoperative Care Unit and later transferred to the ward. After blood collection on postoperative day 3, patients were discharged. At the 3-month follow-up, additional blood samples were collected. Some patients did not attend follow-up appointments due to personal problems and transportation issues. Clinical improvement in OSAS symptoms was reported by all patients during follow-up.

Surgical planning was individualized according to each patient’s anatomical obstruction site and clinical evaluation findings. Preoperative assessment included physical examination, upper airway evaluation, and polysomnographic analysis. Depending on the localization of obstruction, procedures such as septoplasty, uvulopalatopharyngoplasty, tongue base resection, and tongue base suspension were performed either alone or in combination.

All operations were conducted under general anesthesia using standardized anesthetic protocols. Intraoperative monitoring included electrocardiography, pulse oximetry, noninvasive blood pressure monitoring, and capnography. Perioperative oxygen saturation and hemodynamic parameters were maintained within normal limits. Postoperatively, all patients were observed in the postoperative care unit and subsequently followed in the otorhinolaryngology ward according to institutional protocols. Standard postoperative analgesia and supportive care were provided to all participants.

### 2.4. Blood Sampling and Biochemical Analysis

Venous blood samples were collected at four time points: T1 (pre-induction), T2 (end of surgery), T3 (postoperative day 3), and T4 (postoperative month 3). Samples were centrifuged and stored at −80 °C until analysis.

Biochemical analyses were performed using spectrophotometric methods. TAS and TOS were measured using Erel’s methods. AOPP was determined according to Witko-Sarsat et al. [12], IMA was determined using the method of Bar-Or et al. [13], and TT was determined using the Modified Ellman method [14,15].

### 2.5. Statistical Analysis

Data were analyzed using SPSS v20. Continuous variables were expressed as mean ± standard deviation or median (IQR). Non-parametric tests were used where appropriate. Repeated measures were analyzed using the Friedman test, with post hoc comparisons performed using Bonferroni-adjusted Wilcoxon tests. Correlations were evaluated using Spearman’s rho. A *p*-value < 0.05 was considered statistically significant.

## 3. Results

A total of 67 patients were included; 9 (13.4%) were female and 58 (86.6%) were male. The mean age was 45.3 ± 9 years, the mean weight was 88.5 ± 14 kg, the mean height was 171.4 ± 11 cm, and the mean BMI was 30.5 ± 7, indicating a predominantly overweight population. The mean AHI was 31.97 ± 27, reflecting moderate-to-severe OSAS. In our study, none of the patients smoked, and none had a history of chronic or heavy alcohol use. Only 15 patients reported occasionally consuming alcohol in social settings (Table 1).

Analysis of oxidative stress markers revealed no significant changes in TOS, TAS, AOPP, or IMA levels across the four time points (*p* > 0.05) (Table 2). These findings suggest that surgical intervention did not produce measurable short-term changes in systemic oxidative stress or protein oxidation markers.

In contrast, TT levels demonstrated a statistically significant increase at postoperative month 3 compared to earlier measurements (*p* < 0.05). This increase may reflect a delayed adaptive response in antioxidant capacity, particularly in thiol-based redox systems.

Correlation analysis showed that higher AHI values were associated with increased TOS, AOPP, and IMA levels, indicating that oxidative stress and protein damage are linked to disease severity. Additionally, BMI and body weight were positively correlated with AHI, supporting the role of obesity in OSAS pathophysiology (Table 3 and Table 4).

## 4. Discussion

Oxidative stress represents a fundamental mechanism in the pathophysiology of Obstructive Sleep Apnea Syndrome (OSAS), linking intermittent hypoxia to systemic inflammation and end-organ damage. The present study aimed to evaluate whether surgical correction of upper airway obstruction could modulate oxidative stress parameters and improve antioxidant capacity. The findings demonstrate that, despite clinical improvement in symptoms, surgical intervention did not result in significant short-term changes in most oxidative stress markers, including TOS, TAS, AOPP, and IMA. However, a significant increase in Total Thiol (TT) levels was observed at postoperative month 3, suggesting a partial and delayed adaptive response in antioxidant defense.

The relationship between OSAS and oxidative stress has been well documented in the literature. Intermittent hypoxia leads to excessive production of reactive oxygen species (ROS), which overwhelms endogenous antioxidant systems and results in oxidative damage to cellular macromolecules [3,4]. Previous studies have consistently demonstrated elevated oxidant levels and impaired antioxidant capacity in OSAS patients. Baysal et al. reported increased TOS and decreased TAS levels in individuals with OSAS [16], while Bozkurt et al. observed higher oxidant status without significant changes in antioxidant capacity [17]. Similarly, Abakay et al. found a significant imbalance characterized by increased TOS and decreased TAS [18], and Ozben et al. reported elevated TOS and AOPP levels along with reduced TAS [19]. These findings collectively support the concept that oxidative stress is a key contributor to OSAS-related pathophysiology.

In the present study, a positive correlation was observed between AHI and oxidative stress markers such as TOS, AOPP, and IMA, indicating that disease severity is associated with increased oxidative burden. This finding is consistent with previous reports demonstrating that oxidative stress increases with the severity of OSAS [20,21]. The correlation between AHI and IMA also aligns with earlier studies showing that ischemia-related protein modifications are more pronounced in patients with severe disease [22,23,24]. These results reinforce the role of oxidative stress not only as a consequence of OSAS but also as a marker of disease progression.

Despite these associations, surgical intervention did not significantly alter TOS, TAS, AOPP, or IMA levels within the three-month follow-up period. This lack of significant change suggests that correcting upper airway obstruction alone may not be sufficient to reverse the systemic oxidative imbalance in the short term. Several factors may contribute to this finding. First, oxidative stress in OSAS is multifactorial and influenced by comorbid conditions such as obesity, metabolic syndrome, and chronic inflammation. In this study, body weight and BMI were positively correlated with AHI, highlighting the role of obesity as a contributing factor. Persistent metabolic disturbances may continue to drive oxidative stress even after surgical treatment.

Second, the duration of follow-up may not have been sufficient to detect significant biochemical changes. Oxidative damage accumulates over time, and reversal of these processes may require longer periods of sustained improvement in oxygenation and metabolic status. Previous studies evaluating CPAP therapy have shown that improvements in oxidative stress markers may occur gradually and are dependent on treatment adherence and duration [7,8,9,10]. A similar delayed effect may be expected following surgical intervention.

Third, the heterogeneity of surgical procedures may have influenced the results. Different surgical techniques target various anatomical sites and may differ in their effectiveness in reducing airway obstruction and hypoxic episodes. Although control blood samples were taken after 3 months to minimize the effect of the surgical procedure on oxidative stress parameters, it is not known with certainty whether the effect of the surgery on oxidative stress parameters completely disappeared after three months. Without postoperative polysomnographic data, it is difficult to quantify the degree of improvement in AHI and its relationship to oxidative stress markers.

One of the most notable findings of this study was the significant increase in TT levels at postoperative month 3. Thiol groups are critical components of the body’s antioxidant defense system, acting as scavengers of free radicals and maintaining redox balance. Reduced thiol levels have been associated with increased oxidative stress in various conditions [14,15]. Üstündağ et al. reported decreased TT levels in pregnant women with OSAS [25], suggesting impaired antioxidant capacity in this population.

The observed increase in TT levels in the present study may reflect a compensatory or adaptive response to reduced oxidative stress following surgical treatment. Unlike other markers that reflect immediate oxidative damage, thiol levels may represent a more dynamic and responsive component of the antioxidant system. The delayed increase suggests that recovery of antioxidant capacity may occur gradually and may be one of the earliest biochemical signs of improvement following surgery.

The discrepancy between TT and other oxidative markers may also indicate that different components of the oxidative stress system respond differently to intervention. While global oxidant and antioxidant levels (TOS and TAS) remained unchanged, the increase in TT may suggest selective improvement in thiol-based antioxidant mechanisms. This finding highlights the importance of evaluating multiple biomarkers to obtain a comprehensive understanding of oxidative status.

From a clinical perspective, these results suggest that surgical treatment of OSAS, although effective in improving symptoms, may not be sufficient to normalize oxidative stress in the short term. This has important implications for patient management. A multimodal approach that includes lifestyle modifications such as weight loss, regular exercise, and dietary changes may be necessary to achieve meaningful reductions in oxidative stress. Additionally, pharmacological interventions targeting oxidative pathways, such as statins or renin–angiotensin system inhibitors, may provide further benefits.

The findings of this study should be interpreted in light of its limitations. The relatively short follow-up period limits the ability to assess the long-term effects of surgery on oxidative stress. The absence of postoperative polysomnography prevents objective evaluation of improvements in sleep parameters. Furthermore, the sample size, although adequate for statistical analysis, may not have been sufficient to detect subtle biochemical changes. Finally, the inclusion of different surgical procedures introduces variability that may have influenced the outcomes.

Despite these limitations, this study provides valuable insights into the biochemical effects of OSAS surgery. To our knowledge, it is one of the few studies to simultaneously evaluate TOS, TAS, AOPP, IMA, and TT in the perioperative period. The findings contribute to the growing body of evidence suggesting that oxidative stress in OSAS is complex and not easily reversible with a single intervention.

Future research should focus on longer follow-up periods, larger patient populations, and standardized surgical approaches. Studies combining surgical treatment with lifestyle and pharmacological interventions may provide a more comprehensive understanding of how to effectively reduce oxidative stress in OSAS patients. Additionally, incorporating postoperative polysomnographic data would allow for a more precise correlation between clinical improvement and biochemical changes.

In conclusion, while surgical treatment addresses the anatomical component of OSAS, its impact on oxidative stress appears to be limited in the early postoperative period. The observed increase in TT levels suggests a potential adaptive improvement in antioxidant capacity, but this alone may not be sufficient to restore redox balance. These findings underscore the need for a comprehensive, multidisciplinary approach to the management of OSAS, targeting both mechanical obstruction and systemic metabolic dysfunction.

The absence of significant postoperative reductions in TOS, TAS, AOPP, and IMA levels may indicate that biochemical recovery following OSAS surgery occurs more slowly than symptomatic improvement. In addition, some patients in our study were unable to attend the 3-month follow-up appointments, which may have affected the changes in oxidative stress parameters. Oxidative stress in OSAS is influenced not only by upper airway obstruction but also by obesity, metabolic dysfunction, chronic inflammation, and lifestyle-related factors. Therefore, surgical correction of airway obstruction alone may not be sufficient to normalize systemic oxidative pathways within a short follow-up period. The increase observed in TT levels may represent an early adaptive antioxidant response, suggesting partial restoration of redox homeostasis after surgery. In addition, the effectiveness of the surgery after the operation may depend on factors such as unmeasured weight changes after the surgery, diet, and physical activity. These findings highlight the multifactorial nature of oxidative stress in OSAS and emphasize the importance of comprehensive postoperative management strategies.

One study highlighted the importance of gender-related differences in the pathophysiology of OSAS. In a review by Moscucci et al., OSAS in women, particularly in the postmenopausal period, was identified as a poorly recognized cardiovascular risk factor where decreased estrogen levels can increase susceptibility to decreased airway muscle tone, oxidative stress, systemic inflammation, and endothelial dysfunction [26]. These findings support the idea that hormonal and metabolic factors can influence oxidative stress pathways independently of upper airway obstruction. Similarly, in the present study, surgical correction of airway obstruction alone was not sufficient to achieve significant improvements in most oxidative stress markers in the early postoperative period. This may indicate that oxidative imbalance in OSAS is driven not only by mechanical obstruction and intermittent hypoxia but also by broader systemic and metabolic mechanisms. Therefore, the persistence of oxidative stress despite surgical treatment observed in our study can be partly explained by factors outside of airway obstruction, including obesity-related inflammation, metabolic dysfunction, and potentially hormonal effects.

Future studies should include larger patient populations, longer postoperative follow-up periods, and standardized surgical procedures to better evaluate the long-term biochemical effects of OSAS surgery. In addition, postoperative polysomnographic findings should be correlated with oxidative stress markers to determine whether biochemical improvement parallels objective respiratory recovery. Comparative studies evaluating surgery, CPAP therapy, and combined treatment approaches may further clarify the relative impact of different therapeutic modalities on oxidative stress and systemic inflammation. Investigation of additional biomarkers related to endothelial dysfunction, inflammatory cytokines, and mitochondrial activity may also improve understanding of the molecular mechanisms underlying OSAS-associated oxidative injury. Although our study did not include any patients with chronic or heavy alcohol use, 15 patients reported occasionally consuming alcohol in social settings. It is unknown whether this has a significant effect on oxidative stress parameters. It is known that alcohol consumption can affect oxidative stress markers, and the fact that alcohol use was not evaluated in our study is a limitation of our research.

## 5. Conclusions

In conclusion, this prospective observational study evaluated the perioperative effects of OSAS surgery on oxidative stress, antioxidant capacity, and oxidative protein modification using multiple biochemical markers. Although significant postoperative changes were not observed in TOS, TAS, AOPP, or IMA levels during the 3-month follow-up period, a significant increase in TT levels was observed, which may suggest adaptive changes in postoperative protein redox regulation. These findings indicate that surgical treatment alone may not immediately reverse systemic oxidative stress in OSAS patients, despite clinical improvement in symptoms.

The study contributes to the limited literature investigating biochemical responses after OSAS surgery and provides preliminary evidence regarding the complex relationship between surgical intervention and oxidative stress pathways. A more comprehensive postoperative approach incorporating weight management, exercise, metabolic control, and lifestyle modification may be necessary to achieve meaningful biochemical improvement. Further multicenter studies with larger cohorts and longer follow-up durations are warranted to clarify the long-term effects of OSAS surgery on oxidative stress and related systemic outcomes.

### Key Message

What is known

Obstructive sleep apnea syndrome (OSAS) is strongly associated with increased oxidative stress, contributing to cardiovascular and metabolic complications.

Surgical interventions for OSAS primarily aim to alleviate upper airway obstruction, yet their biochemical impact on oxidative stress parameters remains unclear in the short term.

What is new

Surgical treatment did not significantly improve most oxidative stress markers (TOS, TAS, AOPP, IMA) within 3 months, indicating limited early biochemical recovery despite clinical improvement.

Total thiol (TT) levels increased postoperatively, suggesting a partial enhancement of antioxidant capacity, although insufficient to normalize oxidative imbalance.

Adjunctive strategies—smoking cessation, exercise, weight management, diet modification, and metabolic control—may be required to achieve meaningful reductions in oxidative stress, highlighting the need for a multimodal approach.

## Figures and Tables

**Table 1:** Distribution of Some Characteristics of the Participants.

Variables (n = 67)	
Gender	*n*/%
Female	9/13.4
Male	58/86.6
Alcohol consumption	
No	52/77.6
Yes	15/22.4
Smoking status	
No	67/100
Yes	0/0
Chronic disease status	
None	48/71.6
DM	1/1.5
HT	15/22.4
DM + HT	3/4.5
Continuous variables	
	Mean ± SD
Age	45.3 ± 9.09
Weight	88.5 ± 14.6
Height	171.4 ± 11.8
BMI	30.5 ± 7.2

**Table 2:** Oxidative stress and antioxidant markers at different time points before and after OSAS surgery.

Marker	T1	T2	T3	T4	*p* Value
TOS (µmol/L)					χ^2^ = 2.055, *p* = 0.810
Median (IQR)	5.01 (3.76–7.62)	5.01 (3.38–8.10)	5.61 (3.13–8.41)	6.55 (4.48–9.40)	
Mean ± SD (n)	6.14 ± 3.2 (62)	6.44 ± 5.4 (63)	6.65 ± 4.3 (51)	7.5 ± 3.9 (40)	
TAS (mmol/L)					χ^2^ = 5.714, *p* = 0.353
Median (IQR)	2.10 (1.97–2.26)	2.15 (1.89–2.30)	2.18 (1.97–2.31)	1.99 (1.65–2.20)	
Mean ± SD (n)	2.07 ± 0.3 (62)	2.00 ± 0.4 (63)	2.08 ± 0.3 (51)	1.81 ± 0.4 (40)	
AOPP (µmol/L)					χ^2^ = 12.097, *p* = 0.056
Median (IQR)	20.05 (13.8–30.1)	18.10 (11.6–28.3)	17.26 (12.8–30.0)	24.09 (16.6–38.2)	
Mean ± SD (n)	25.8 ± 17 (61)	23.1 ± 16 (60)	24.1 ± 17 (48)	31.2 ± 20 (36)	
IMA (IU/mL)					χ^2^ = 3.60, *p* = 0.308
Median (IQR)	92.70 (82.8–101.3)	82.25 (82.6–98.3)	91.90 (83.9–101.6)	92.75 (82.0–112.1)	
Mean ± SD (n)	95.03 ± 16 (62)	93 ± 16 (62)	93 ± 14 (52)	98 ± 23 (40)	
TT (µmol/L)					χ^2^ = 16.650, *p* = 0.001
Median (IQR)	652.14 ^a^ (539–652)	615.53 ^b^ (492–778)	592.42 ᶜ (502–693)	794.95 ^a,b,c^ (655–1024)	
Mean ± SD (n)	668.8 ± 217 (61)	686.4 ± 282 (62)	617.3 ± 173 (52)	853 ± 275 (38)	

Different superscript letters (^a^, ^b^, ^c^) indicate statistically significant differences between the corresponding time points.

**Table 3:** Correlation relationships between AHI and biomarkers, weight and BMI.

Variables (n = 67)	Spearman’s Rho	*p*	% 95 Confidence İntervals
AHI-TAS1	−0.214	>0.05	−0.44–0.46
AHI-TOS1	0.273	=0.03	0.01–0.49
AHI-AOPP1	0.328	=0.01	0.07–0.5
AHI-TİYOL1	−0.83	>0.05	−0.33–0.18
AHI-IMA1	0.331	=0.009	0.08–0.54
AHI-KİLO	0.363	=0.003	0.12–0.56
AHI-BMI	0.298	=0.01	0.05–0.85

A positive correlation was found between participants’ TOS1, AOPP1, IMA1, weight, and BMI values and AHI measurements (*p* < 0.05 for all).

**Table 4:** Types of Operations Performed in the Participants.

Types of Operations Performed (*n* = 67)	* N	%
Septoplasty	50	74.6
Conchaplasty	36	53.7
Uvulopalatopharyngoplasty	50	74.6
Septorhinoplasty	1	1.5
Tongue base suspension	25	37.3
Soft palate remodeling	9	13.4

* Since participants underwent multiple surgical procedures during the same operation, the number of surgical procedures exceeded the number of patients.

## Data Availability

The data that support the findings of this study are available from the corresponding author upon reasonable request.
